# Challenge Accepted: Retinopathy of Prematurity (ROP) During COVID-19

**DOI:** 10.7759/cureus.35260

**Published:** 2023-02-21

**Authors:** Sandip K Sahu, Priyadarshini Mishra

**Affiliations:** 1 Ophthalmology, All India Institute of Medical Sciences, Bhubaneswar, Bhubaneswar, IND

**Keywords:** rop, challenge, covid-19, screening, retinopathy of prematurity

## Abstract

The World Health Organization has identified retinopathy of prematurity (ROP) as one of the emerging causes of preventable childhood blindness in developing and middle-income countries. It is becoming a major public health problem in developing countries like India and China. ROP blindness in India is increasing due to the highest number of preterm births in the world, suboptimal neonatal care, lack of awareness, screening programs and treatment not in place, and increasing numbers of neonatal intensive care units (NICUs) and special newborn care units (SNCUs) opening all over the country without appropriate ophthalmic care. On top of it, heavier and late preterm babies are developing severe ROP due to the variable quality of neonatal services and insufficient optimal eye care in the NICU and SNCU. The situation become more precarious during the coronavirus disease 2019 (COVID-19) pandemic, and it became a challenge to motivate health workers and parents to keep the screening and treatment of ROP babies in place.

## Introduction

Retinopathy of prematurity (ROP), a proliferative retinopathy among preterm/premature infants, is emerging as the leading cause of blindness in developing and middle-income countries [1]. It is becoming a major public health problem in India. The main factors for the increase in the incidence of ROP blindness in India are the highest number of preterm births in the country globally, suboptimal neonatal care, lack of awareness, inadequate screening and treatment programs, and increasing numbers of neonatal intensive care units (NICUs) and special newborn care units (SNCUs) opening all over the country [2,3].

## Technical report

Indication

According to the All India Ophthalmological Society-Indian Journal of Ophthalmology consensus statement regarding preferred practices, ophthalmic emergencies are determined by the ophthalmologists’ judgment of the potential risk to vision, eye, and life and the impact on the patient’s quality of life, if the disease is left untreated [4]. In view of this guideline, ROP screening and management come under ophthalmic emergencies. Screening and treating neonates with ROP were challenging during the coronavirus disease 2019 (COVID-19) pandemic period. Because India is the country with the maximum number of premature births and the birth rate remained nearly unchanged during the COVID-19 pandemic, the number of ROP cases also remained the same as before. In addition, there is probable congenital severe acute respiratory syndrome coronavirus 2 (SARS-CoV-2) infection in a neonate born to women with active SARS-CoV-2 infection [5]. The risk of transmission was present during the screening and treatment in the newborn intensive care unit.

Problems

During the COVID-19 pandemic period, restrictions were imposed and hospitals were shut down, which led to an obvious shortage of neonatal ICU beds. The number of NICU doctors and staff was reduced greatly because of the imposed quarantine restrictions. At such times, providing optimum care was a challenge. Moreover, parents were not able to bring the neonates to the hospital for timely screening owing to travel restrictions, economic fallout, and miscommunications regarding the operability of hospitals. All these factors contributed to a delay in diagnosis and treatment. Keeping this in view, proliferative retinopathy among preterm infants may lead to the cause of blindness or severe low vision, or other ocular morbidities, if remain unaddressed. It became a challenge to motivate health workers and parents with appropriate accessories to keep the screening and treatment for ROP babies in place.

Preventive measures

**Figure 1:**
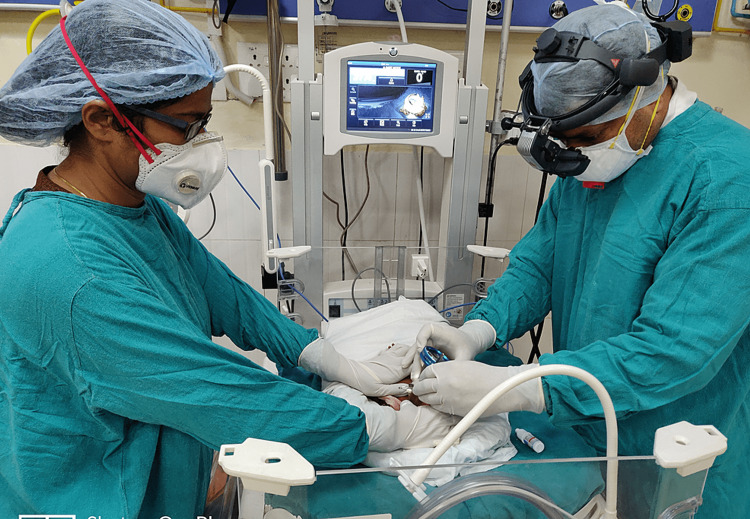
Screening is done with a surgical gown + N-95 mask + shoe cover + cap + surgical gloves

**Figure 2:**
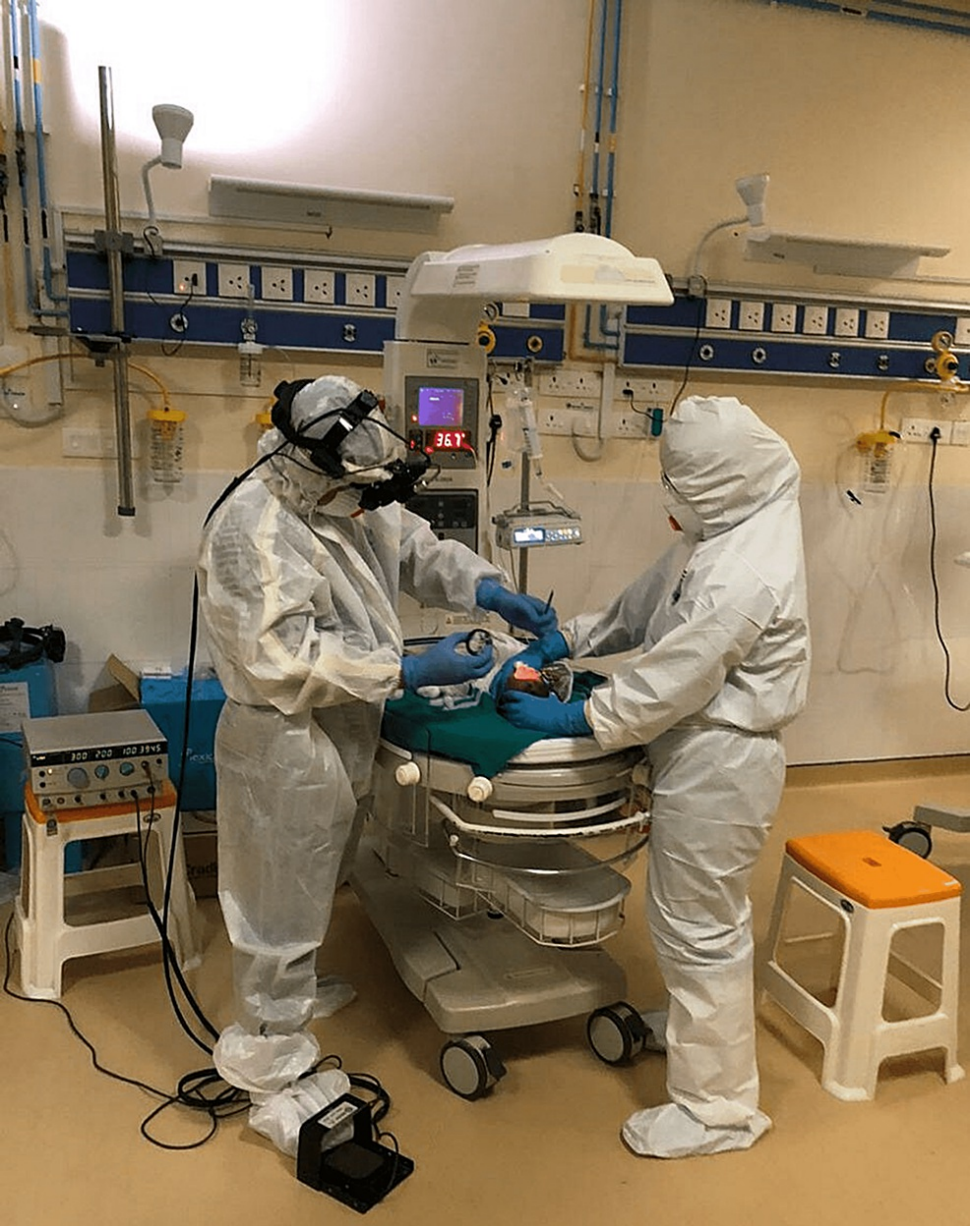
Laser treatment is done with ideal PPE (Type A) composed of a Hazmat suit + N-95 mask + visor + shoe cover + gloves PPE: personal protective equipment

To keep up with the double battle, we ensure a backup screening group, which could take over in case the first group needed to be quarantined. The screening group was minimally exposed to outdoors and other ocular emergencies. The screening was performed with surgical gown + N-95 mask + shoe cover + cap + surgical gloves (Figure 1), whereas laser treatment was performed with ideal Personal protective equipment (PPE) (Type A) composed of Hazmat suit + N-95 mask + visor + shoe cover + gloves (Figure 2). Crossover screenings were encouraged for patients having travel difficulties, and such patients were referred to centers with the facility for ROP screening located in proximity to their residences. The patients who required treatment or were scheduled to undergo check-ups over short periods were kept in the NICU under the supervision of experienced staff and doctors trained in neonatal care. The follow-up screening protocol was modified with longer follow-up periods for infants with a gestational age of >28 weeks, birth weight of >1200 grams, weight gain at par with normal neonates of that age group, and lesions of type-2 ROP.

Expected outcomes

All these above steps help us keep the screening and treatment for ROP in place and the number of babies screened and treated for ROP was almost the same as the normal days in our hospital. In our institution, during the pre-lockdown period and lockdown periods, 17, 12, and 16 neonates were screened in March, April, and May 2020, respectively. With the upliftment of lockdown and transport restrictions in June 2020, 26 neonates were screened, and with partial lockdown in July 2020, 14 neonates were screened, whereas, in the subsequent two months, 36 neonates were screened. During that period, 121 neonates (242 eyes) were screened, and three neonates (5 eyes) underwent laser treatment.

Complications and limitations

In the above process, lots of our health workers were infected with COVID-19. Parents and babies have faced a few difficulties due to lockdown and restrictions and a few babies were lost to follow-up.

## Discussion

ROP is a potentially permanent blinding disease, which can be prevented by timely screening and management. The COVID-19 pandemic has significantly hampered the health delivery system. We try to manage the ROP screening and management during the pandemic with lots of hurdles. Keeping an eye on the pandemic in future digital innovations, including artificial intelligence (AI), 5th generation (5G) telecommunication and the Internet of Things (IoT) can create an inter-dependent system offering opportunities to develop new models of eye care addressing the challenges of COVID-19 and beyond [6]. Parents could able to upload text, audio, video, and images and able to conduct a conversation with an ophthalmologist via the teleophthalmology platform in Wuhan Children’s hospital in Wuhan, China, from January to October 2020 during the pandemic [7]. For ROP screening, it may be quite difficult, as the physical presence of ophthalmologists and ophthalmic-trained nurses is needed.

For safe, secure, effective, and compatible ROP screening and management, the following are required: optimal use of technology, measures to minimize the risk of transmission of infections, and reduce multiple screening for lower-risk babies by keeping an eye on parameters like weight gain per day, kangaroo mother care (KMC), urine output and other developmental milestones, which can be reported by parents. The use of risk-stratification algorithms should be considered to reduce examinations for low-risk infants [8]. Concentrating on high-risk babies and timely intervention is the key point for optimal screening and management in pandemic-like situations.

## Conclusions

We were in a catch-22 situation. Clear national or international consensus and protocols for clinical practice and ensuring the protection of health professionals, environmental care, instrument cleaning, and appropriate custom-designed instruments are the rule of thumb to overcome these situations.

## References

[1] Dogra MR, Katoch D (2017). Retinopathy of prematurity: an emerging and evolving challenge. Indian J Ophthalmol.

[2] Vinekar A, Dogra MR, Sangtam T, Narang A, Gupta A (2007). Retinopathy of prematurity in Asian Indian babies weighing greater than 1250 grams at birth: ten year data from a tertiary care center in a developing country. Indian J Ophthalmol.

[3] Shah PK, Narendran V, Kalpana N (2012). Aggressive posterior retinopathy of prematurity in large preterm babies in South India. Arch Dis Child Fetal Neonatal Ed.

[4] Sengupta S, Honavar SG, Sachdev MS (2020). All India Ophthalmological Society - Indian Journal of Ophthalmology consensus statement on preferred practices during the COVID-19 pandemic. Indian J Ophthalmol.

[5] Kirtsman M, Diambomba Y, Poutanen SM (2020). Probable congenital SARS-CoV-2 infection in a neonate born to a woman with active SARS-CoV-2 infection. CMAJ.

[6] Li JO, Liu H, Ting DS (2021). Digital technology, tele-medicine and artificial intelligence in ophthalmology: a global perspective. Prog Retin Eye Res.

[7] Guo Z, Ma N, Wu Y (2021). The safety and feasibility of the screening for retinopathy of prematurity assisted by telemedicine network during COVID-19 pandemic in Wuhan, China. BMC Ophthalmol.

[8] Mantagos IS, Wu C, Griffith JF (2021). Retinopathy of prematurity screening and risk mitigation during the COVID-19 pandemic. J AAPOS.

